# Adrenergic Blockade Bi-directionally and Asymmetrically Alters Functional Brain-Heart Communication and Prolongs Electrical Activities of the Brain and Heart during Asphyxic Cardiac Arrest

**DOI:** 10.3389/fphys.2018.00099

**Published:** 2018-02-13

**Authors:** Fangyun Tian, Tiecheng Liu, Gang Xu, Duan Li, Talha Ghazi, Trevor Shick, Azeem Sajjad, Michael M. Wang, Peter Farrehi, Jimo Borjigin

**Affiliations:** ^1^Department of Molecular and Integrative Physiology, University of Michigan, Ann Arbor, MI, United States; ^2^Department of Neurology, University of Michigan, Ann Arbor, MI, United States; ^3^Neuroscience Graduate Program, University of Michigan, Ann Arbor, MI, United States; ^4^Cardiovascular Center, University of Michigan, Ann Arbor, MI, United States; ^5^Veterans Administration Ann Arbor Healthcare System, Ann Arbor, MI, United States; ^6^Department of Internal Medicine-Cardiology, University of Michigan, Ann Arbor, MI, United States; ^7^Michigan Center for Integrative Research in Critical Care, University of Michigan, Ann Arbor, MI, United States

**Keywords:** atenolol, phentolamine, autonomic nervous system, coherence, directional connectivity, asphyxic cardiac arrest

## Abstract

Sudden cardiac arrest is a leading cause of death in the United States. The neurophysiological mechanism underlying sudden death is not well understood. Previously we have shown that the brain is highly stimulated in dying animals and that asphyxia-induced death could be delayed by blocking the intact brain-heart neuronal connection. These studies suggest that the autonomic nervous system plays an important role in mediating sudden cardiac arrest. In this study, we tested the effectiveness of phentolamine and atenolol, individually or combined, in prolonging functionality of the vital organs in CO_2_-mediated asphyxic cardiac arrest model. Rats received either saline, phentolamine, atenolol, or phentolamine plus atenolol, 30 min before the onset of asphyxia. Electrocardiogram (ECG) and electroencephalogram (EEG) signals were simultaneously collected from each rat during the entire process and investigated for cardiac and brain functions using a battery of analytic tools. We found that adrenergic blockade significantly suppressed the initial decline of cardiac output, prolonged electrical activities of both brain and heart, asymmetrically altered functional connectivity within the brain, and altered, bi-directionally and asymmetrically, functional, and effective connectivity between the brain and heart. The protective effects of adrenergic blockers paralleled the suppression of brain and heart connectivity, especially in the right hemisphere associated with central regulation of sympathetic function. Collectively, our results demonstrate that blockade of brain-heart connection via alpha- and beta-adrenergic blockers significantly prolonged the detectable activities of both the heart and the brain in asphyxic rat. The beneficial effects of combined alpha and beta blockers may help extend the survival of cardiac arrest patients.

## Introduction

Fatal cardiac arrest affects more than 400,000 Americans each year (Chugh et al., 2008; Stecker et al., 2014). Despite decades of intensive research efforts, survival rate from cardiac arrest is only about 5% (Nolan et al., 2012; Stecker et al., 2014). Sudden death occurs in patients with cardiovascular disease as well as those with no known history of heart disease, including individuals with ischemic stroke, traumatic brain injury, epilepsy, chronic obstructive pulmonary disease, and asphyxia (Samuels, 2007; Sörös and Hachinski, 2012; Israel, 2014; Lahousse et al., 2015). Unfortunately, current studies of cardiac arrest are largely focused on the cardiovascular pathology and methods of cardiac resuscitation; very little attention has been given to the role of the brain prior to the arrest of the heart.

Recent studies from our laboratory demonstrate that the brain plays a key role in cardiac arrest. Experimental cardiac arrest (Borjigin et al., 2013) and asphyxic cardiac arrest (Li et al., 2015a) both lead to a rapid surge of functional connectivity (coherence) and effective connectivity in the dying brain. The marked surge of cortical coherence (CCoh) and directional cortical connectivity (CCon) paralleled dramatically increased release of a set of core neurotransmitters in the brain (Li et al., 2015a). Importantly, asphyxia activates a delayed surge of cortex-heart coupling, a novel form of communication measured by corticocardiac coherence (CCCoh) and directional corticocardiac connectivity (CCCon) (Li et al., 2015a). We hypothesized that the stimulated brain functions to resuscitate the heart internally by activating the sympathetic nervous system (Li et al., 2015a), the main mechanism thought to lead to sudden cardiac arrest in high risk patients (Samuels, 2007; Dhalla et al., 2010). Past studies have shown that experimental cardiac arrest stimulates excessive neural release of catecholamines leading to fatal ventricular arrhythmias (Foley et al., 1987; Borovsky et al., 1998; Dhalla et al., 2010). Consistent with these reports, a marked surge of cardiac sympathetic activity was reported in patients with sustained ventricular arrhythmias (Meredith et al., 1991). These data demonstrate that increased cardiac sympathetic activity is causally linked with cardiac failures (Samuels, 2007; Taggart et al., 2011; Silvani et al., 2016).

Beta blockers (blockers of beta-adrenergic receptors) are widely used to manage cardiac arrhythmias and to prevent a second myocardial infarction in heart attack patients (Yusuf et al., 1985; Freemantle et al., 1999; Bourque et al., 2007) and are shown to reduce the incidence of ventricular fibrillation (VF) after acute myocardial infarction (Rydén et al., 1983; Norris et al., 1984). Despite the mounting evidence for the positive effects of beta blockers to protect diseased hearts, the mechanism by which beta-blockers prevent ventricular arrhythmias is not well-understood (Yusuf et al., 1985; Bourque et al., 2007).

In our earlier study (Li et al., 2015a), surgical blockade of efferent neuronal outflows traveling down the spinal cord below the cervical level 7 (C7) significantly extends the electrical activities of both the heart and the brain in the dying rats. Since sympathetic signals exit the spinal cord below C7 in rats, the beneficial effects of the C7 transection procedure is likely mediated by the blockade of sympathetic impact on the heart. The present study is designed to test if and how pharmacological blockade of the adrenergic receptors of the heart influences the cortical oscillations and changes the dynamics of the brain-heart electrical coupling in CO_2_-mediated asphyxic cardiac arrest model.

The sympathetic nervous system regulates the cardiovascular function via both alpha- and beta-adrenergic receptors. While alpha- and beta-adrenergic receptor blockers are used together to increase efficacy of hypertension treatment (Ram and Kaplan, 1979; Wong et al., 2015), their combined impact on delaying the onset of cardiac arrest has not been evaluated. In this study, we tested the impact of phentolamine (non-selective alpha adrenergic receptor blocker) and atenolol (selective beta-1 adrenergic receptor blocker), alone or combined, on the duration of cortical and cardiac electrical activity, CCoh, CCCoh, and CCCon in asphyxic rats.

## Materials and methods

### Animals

Inbred male Fischer 344 rats from Harlan were acclimatized in our housing facility for at least 1 week before surgical implantation of electrodes. After implantation, rats were allowed to recover for at least 1 week before online recording. All experiments were conducted using adult rats (300–400 g) maintained on a light: dark cycle of 12: 12 h and provided with *ad libitum* food and water. This study was carried out in accordance with the recommendations of the University of Michigan Committee on Use and Care of Animals. The protocol was approved by the University of Michigan Committee on Use and Care of Animals.

### Electrode implantation and configuration

Rats were implanted with electrodes for ECG and EEG recording under surgical anesthesia (1.8% (vol/vol) isoflurane). ECG was recorded through flexible and insulated multi-stranded wires (Cooner Wire) inserted into the subcutaneous muscles flanking the heart. EEG was recorded through screw electrodes implanted bilaterally on the frontal [anteroposterior (AP): + 3.0 mm; mediolateral (ML): ± 2.5 mm, bregma], parietal (AP: −3.0 mm; ML ± 2.5 mm, bregma), and occipital (AP: −8.0 mm; ML: ± 2.5 mm, bregma) cortices. A nose electrode was used as the EEG reference (Borjigin et al., 2013). The ECG and EEG electrodes were interfaced with two six-pin pedestals (Plastics One) and secured on the skull with dental acrylic.

### Signal acquisition

Before data collection, rats were acclimatized overnight in the recording chamber. ECG and EEG were recorded using Grass Model 15LT physiodata amplifier system (15A54 Quad amplifiers, Astro-Med, Inc.) interfaced with BIOPAC MP-150 data acquisition unit and AcqKnowledge software (version 4.1.1, BIOPAC Systems, Inc.). Signals were filtered between 0.1 and 300 Hz and sampled at 1,000 Hz. ECG and EEG recordings were initiated consistently at 10:00 a.m. to control for circadian factors. The rats were divided into 4 groups. Baseline signals were recorded for at least 30 min for all the rats. Then each group of rats received either saline (*n* = 10), phentolamine (10 mg/kg, *n* = 7), atenolol (10 mg/kg, *n* = 8), or phentolamine plus atenolol (10 mg/kg, 10 mg/kg, *n* = 11). Thirty minutes after drug injection, cardiac arrest was induced by inhalation of CO_2_ (30%) for 2 min. Recording was continued for another 30 min after asphyxia.

### Analysis of RR interval (RRI) and cardiac arrhythmias

To analyze the RRI (the time intervals between the R-peaks of two adjacent heartbeats), baseline drift correction was first implemented using second-order Butterworth high-pass filtering with a cutoff frequency at 1 Hz (butter.m and filtfilt.m in Matlab Signal Processing Toolbox; MathWorks Inc.). R-peak of ECG signals was then detected using variable threshold method (Kew and Jeong, 2011). Specifically, an amplitude threshold in each nonoverlapping 1 s epoch was applied to select the candidates for R peaks, which can be verified only if the RRI value exceeds a predefined threshold. In this study, the interval threshold was selected as half of the median value of the RRI values in the last 1 s epoch. The automatically detected R peaks were manually validated through a custom user interface developed in Matlab (MathWorks Inc.). To analyze the number and types of cardiac arrhythmias, ECG signals were examined and cardiac arrhythmias were manually labeled using a custom user interface developed in Matlab (MathWorks Inc.).

### Construction of Electrocardiomatrix (ECM)

The ECM is designed to facilitate the visualization of RRI, the amplitude, and the morphology of ECG signals. For construction of ECM (Li et al., 2015b), a window centered on the detected ECG R peaks (for example, from 0.1 s to 0.3 s, with 0 corresponding to the time of R-peak) was extracted from the ECG signal after baseline drift correction. All ECG windows were sorted according to the order of R-peak time and then plotted as parallel colored lines to form a colored rectangular image. The intensity of ECG signal was denoted on *z*-axis, with warmer color indicates positive peaks with higher voltage, while cooler color indicates negative peaks with lower voltage. The color scheme could be adjusted according to the need.

### Analysis of CCoh and CCCoh

The coherence between six EEG channels (CCoh) or between one ECG and each of the six EEG channels (CCCoh) were measured by amplitude squared coherence (*C*_*xy*_(*f*)) (mscohere.m in Matlab Signal Processing Toolbox; MathWorks Inc.), which is a coherence estimate of the input signals x and y using Welch's averaged, modified periodogram method. The magnitude squared coherence *C*_*xy*_(*f*) is a function of frequency with values between 0 and 1 that indicates how well signal x corresponds to signal y at each frequency.

(1) $$\begin{array}{r} C_{xy}\left(f\right)=\frac{|P_{xy}\left(f\right){|}^{2}}{P_{xx}\left(f\right)P_{yy}\left(f\right)},0\leq C_{xy}\left(f\right)\leq 1 \end{array}$$

where *P*_*xx*_(*f*) and *P*_*yy*_(*f*) are the power spectral density of x and y, and *P*_*xy*_(*f*) is the cross power spectrum spectral density.

In current study, electrophysiological signals were first segmented into 2 s epochs with 1 s overlap. The magnitude squared coherence was then calculated at each epoch and frequency bin (from 0.5 to 250 Hz). Before coherence analysis, a notch filter was used to remove the 60 Hz artifact and its possible super-harmonics. For each rat, the mean coherence among 15 pairs of six EEG channels (**Figure 2A**), the mean coherence among one ECG and six EEG channels (**Figure 5A**), as well as the coherence between one ECG and each of the six EEG channels (6 pairs; **Figure 6A**) were calculated and plotted for frequencies from 0.5 to 250 Hz. The mean and standard deviation (SD) of coherence between one ECG and six EEG channels (**Figure 5B**) and the coherence between one ECG and each of the six EEG channels (**Figure 6B**) were calculated for frequencies from theta to gamma 1 (theta: 5–10 Hz, alpha: 10–15 Hz, beta: 15–25 Hz, and gamma 1: 25–55 Hz).

### Analysis of CCCon

The directional connectivity between the heart and brain (or between one ECG and each of the six EEG channels, CCCon) was measured by a modified (Li et al., 2015a) Normalized Symbolic Transfer Entropy (NSTE) method (Lee et al., 2009), which is a nonlinear and model-free estimation of directional functional connection based on information theory. STE denotes the amount of information provided by the additional knowledge from the past of the source signal *X* (*X*^*P*^) in the model describing the information between the past *Y* (*Y*^*P*^) and the future *Y* (*Y*^*F*^) of the target signal *Y*, which is defined as follows:

(2) $$\begin{array}{r} STE_{X\to Y}=I\left(Y^{F};X^{P}|Y^{P}\right)=H\left(Y^{F}|Y^{P}\right)-H\left(Y^{F}|X^{P},Y^{P}\right) \end{array}$$

where *H*(*Y*^*F*^|*Y*^*P*^) is the entropy of the process *Y*^*F*^conditional on its past. Each vector for *Y*^*F*^, *X*^*P*^ *and Y*^*P*^is a symbolized vector point. The potential bias of STE was removed with a shuffled data, and the unbiased STE is normalized as follows:

(3) $$\begin{array}{r} NSTE_{X\to Y}^{\;}=\frac{STE_{X\to Y}-{STE_{X\to Y}^{Shuffled}}_{\;}}{H\left(Y^{F}|Y^{\;}\right)}\in \left[0,\;1\right] \end{array}$$

where $STE_{X\to Y}^{Shuffled}=H\left(Y^{F}|Y^{P}\right)-H\left(Y^{F}|X_{Shuff}^{P},Y^{P}\right)$. $X_{Shuff}^{P}\;$is a shuffled data created by dividing the data into sections and rearranging them at random. Therefore, NSTE is normalized STE (dimensionless), in which the bias of STE is subtracted from the original STE and then divided by the entropy within the target signal, *H*(*Y*^*F*^|*Y*^*P*^).

For CCCon, the feedback (FB) connectivity (*NSTE_EEG→EKG_*) was calculated by averaging NSTE over six pairs of EEG channels to ECG channel, which are defined as follows:

(4) $$\begin{array}{r} {\bar{NSTE}}_{EEG\to EKG}=\frac{1}{n_{EEG}}\overset{n_{EEG}}{\underset{i\;=\;1}{\sum }}NSTE_{i\to EKG} \end{array}$$

where *n*_*EEG*_ = 6. The feedforward (FF) connectivity (${\bar{NSTE}}_{EKG\to EEG}$) from the ECG to six EEG channels is vice versa.

Specifically, we first filtered EEG and ECG signals into 4 frequency bands (theta, alpha, beta, and gamma 1) and then segmented the filtered signals into 2 s long epochs with 1 s overlapping. The mean CCCon (${\bar{NSTE}}_{EEG\to EKG}$ and ${\bar{NSTE}}_{EKG\to EEG}$) were sequentially calculated for each epoch and each frequency band. Three parameters: embedding dimension (*d*_*E*_), time delay (τ), and prediction time (δ), were required in the calculation. In this study, we selected the parameter setting that could yield maximum $NSTE_{X\to Y}^{\;}$ by fixing the embedding dimension (*d*_*E*_) at 3, and optimizing prediction time δ (from 1 to 50, corresponding to 1–50 ms with the sampling frequency of 1,000 Hz) and time delay τ (1–300 ms). The same procedure was used to calculate $NSTE_{Y\to X}^{\;}$, provided that the information between two signals is transferred through different neuronal pathway. The mean and SD of the averaged CCCon for all 4 frequencies among six EEG channels (**Figure 8**) and that for each of the six EEG channels (6 pairs; **Figure 9**) were calculated and plotted.

### Statistical analysis

**Figure 1:**
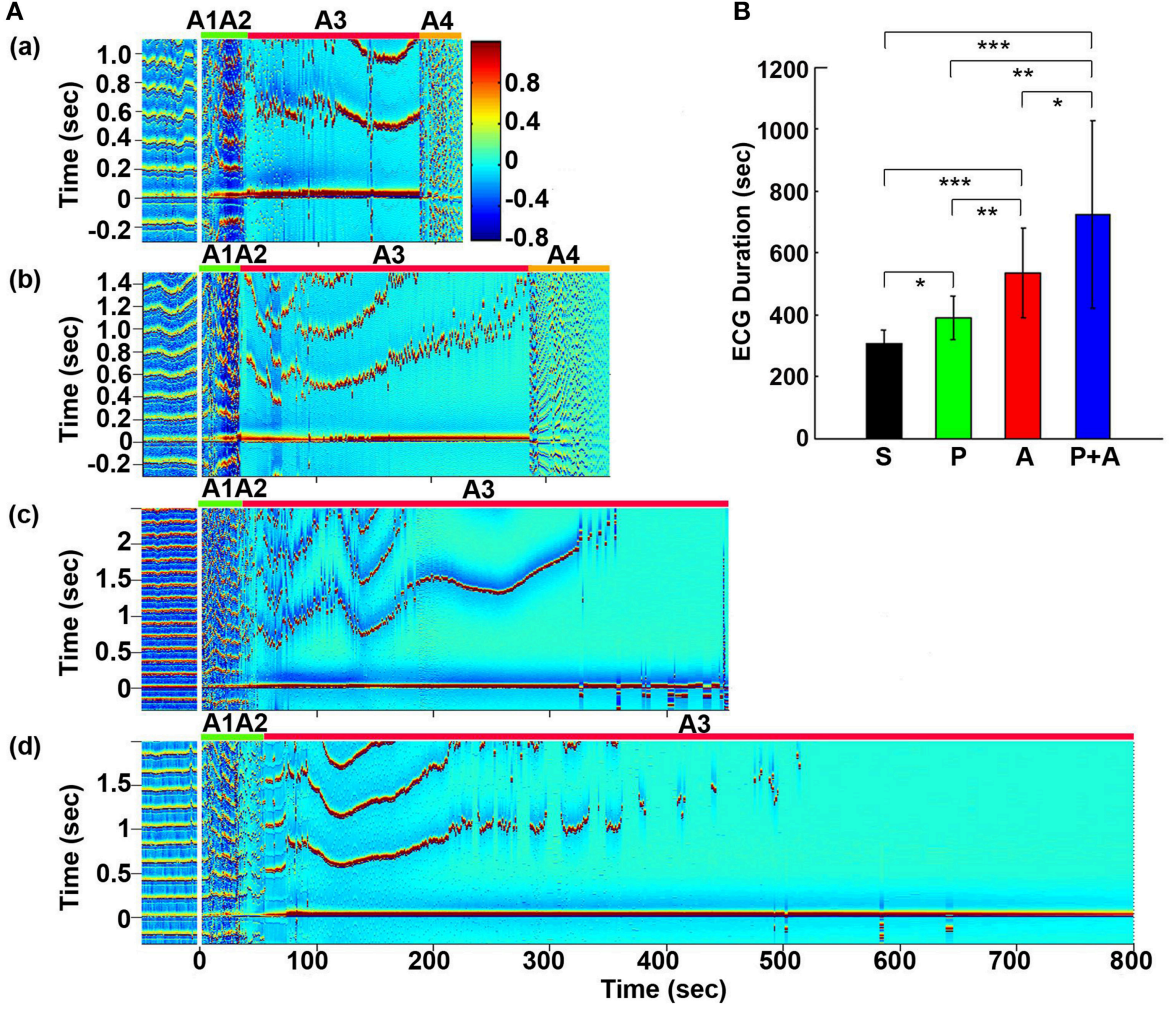
Adrenergic blockade prolongs cardiac survival. **(A)** Electrocardiomatrix (ECM) display of ECG signals before (50 s) and after asphyxia in four groups of rats: **(a)** saline, **(b)** phentolamine, **(c)** atenolol, and **(d)** phentolamine plus atenolol. *x* axis shows time in seconds, *y* axis shows RR interval (RRI; in seconds), and *z* axis shows signal strength. Warmer color represents higher signal strength. Asphyxia was induced by CO_2_ infusion at time 0 s. **(B)** The mean and SD of ECG signal duration after asphyxia in four groups of rats: S (saline, *n* = 10), P (phentolamine, *n* = 7), A (atenolol, *n* = 8), and P+A (phentolamine plus atenolol, *n* = 11). Significant differences of ECG signal duration among 4 groups of rats are indicated using asterisks. Error bars denote SD (^*^p < 0.05, ^**^p < 0.01, ^***^p < 0.001).

**Figure 2:**
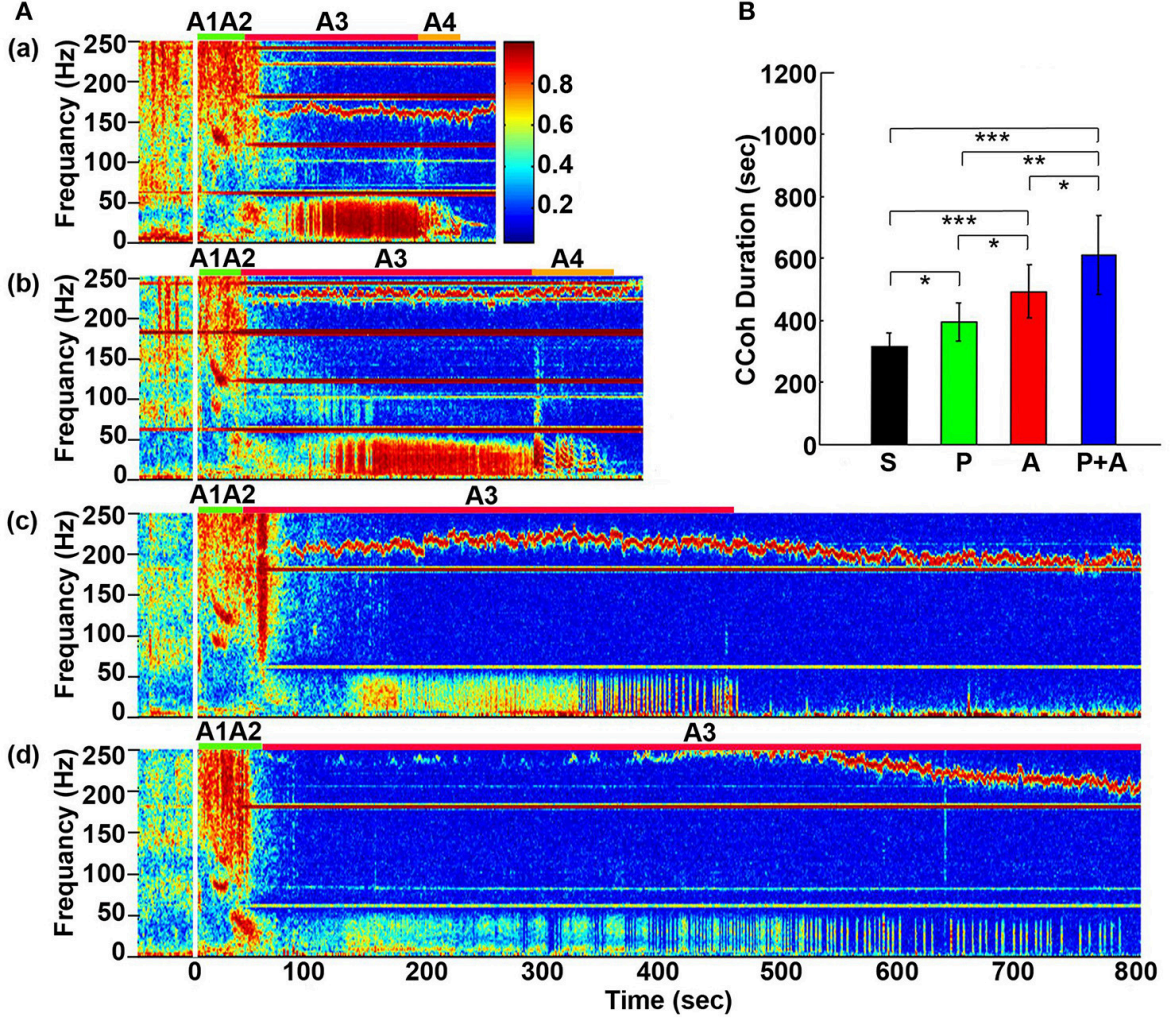
Adrenergic blockade prolongs cortical coherence (CCoh) duration. **(A)** CCoh (averaged over six EEG channels) before (50 s) and after asphyxia in four groups of rats: **(a)** saline, **(b)** phentolamine, **(c)** atenolol, and **(d)** phentolamine plus atenolol. *x* axis shows time, *y* axis shows frequency, and *z* axis shows CCoh. Warmer color represents stronger CCoh. Asphyxia was induced at time 0 s. **(B)** The mean and SD of CCoh duration after asphyxia in four groups of rats: S (saline, *n* = 10), P (phentolamine, *n* = 7), A (atenolol, *n* = 8), and P+A (phentolamine plus atenolol, *n* = 11). Significant differences of CCoh duration among 4 groups of rats are indicated using asterisks. Error bars denote SD (^*^p < 0.05, ^*^^*^p < 0.01, ^***^p < 0.001).

**Figure 3:**
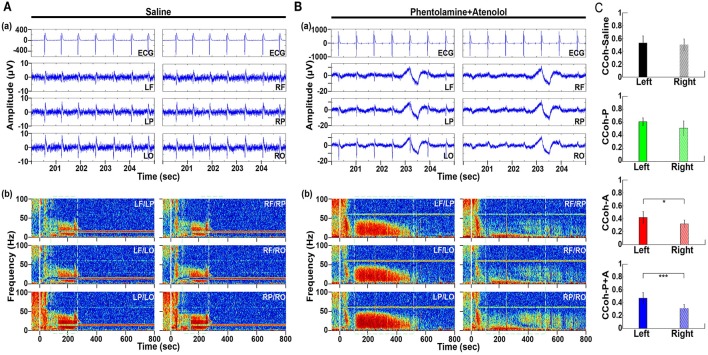
Adrenergic blockade leads to a marked hemispheric asymmetry of cardiac event related potential (CERP) and cortical coherence (CCoh). Raw ECG and EEG data (200–205 s in A3), as well as CCoh raw data (50 s before and 800 s after asphyxia), for each hemispheric channel or channel-pair among the six EEG channels, is shown for one representative control rat **(A)** and a rat received phentolamine plus atenolol **(B)**. The blockers inhibit cardiac event related EEG potentials **(Ba)** and CCoh **(Bb)** specifically on the right hemisphere and the effects are more significant when phentolamine plus atenolol were used together **(C)**. Significant differences of mean pairs of CCoh, calculated for A3 at 5–55 Hz, are indicated using asterisks in **(C)**. LF: left frontal; RF: right frontal; LP: left parietal; RP: right parietal; LO: left occipital; RO: right occipital. Error bars denote SD (^*^p < 0.05, ^***^p < 0.001).

**Figure 4:**
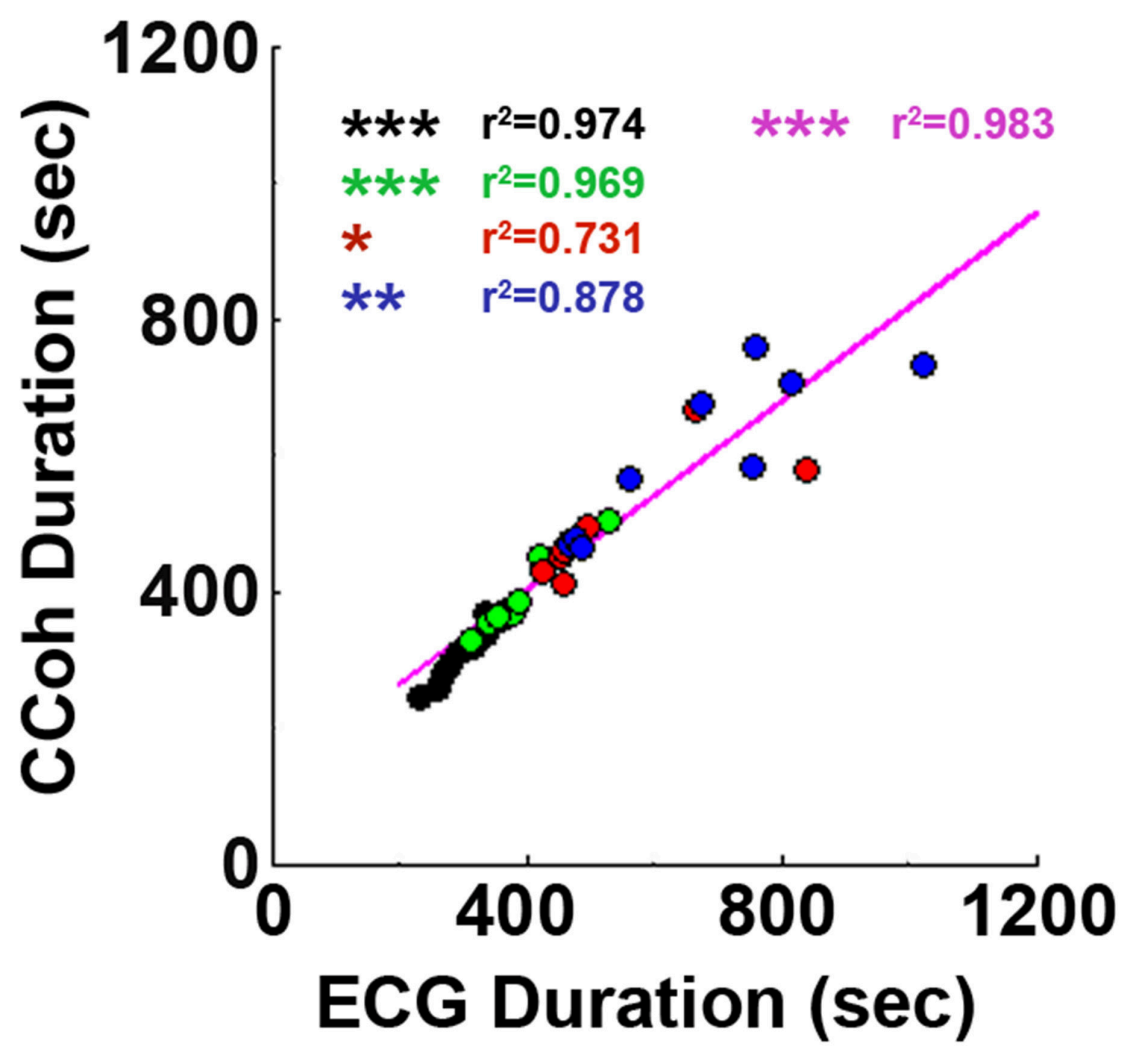
Cardiac survival parallels with cortical coherence (CCoh) duration. Each circle represents data from one rat. Linear regression line was plotted to show the correlation between ECG signal duration and CCoh duration for all four groups of rats. Significant correlations between ECG signal duration and CCoh duration are indicated using asterisks. Different color circles represent different treatment groups: saline control in black, phentolamine in green, atenolol in red and phentolamine plus atenolol in blue. The magenta color represents values obtained with all four groups of rats (*n* = 35). (^*^p < 0.05, ^**^p < 0.01, ^***^p < 0.001).

**Figure 5:**
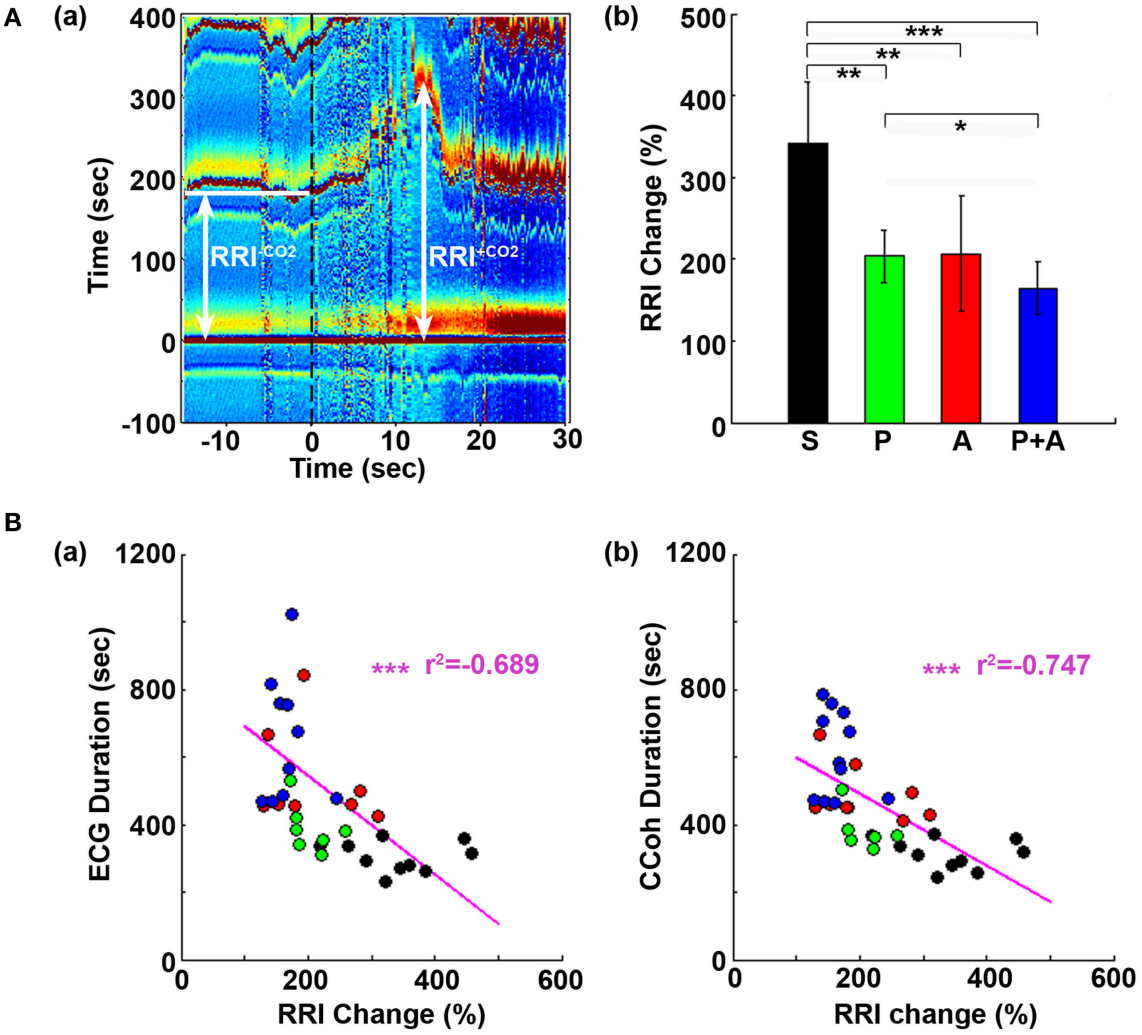
Adrenergic blockade suppresses the initial rise of RR interval (RRI), which negatively correlates with cardiac survival and cortical coherence (CCoh) duration. **(A)** Electrocardiomatrix (ECM) display of ECG signals −15 s before and 30 s after asphyxia for one control rat **(a)**. RRI^−CO2^ represents the averaged baseline RRI (100 s before asphyxia); RRI^+CO2^ indicates the peak amplitude of the first increase of RRI after asphyxia. The mean and SD of RRI change (RRI^+CO2^ × 100/RRI^−CO2^) shows drug-specific changes **(b)**. Significant differences of RRI among the rats are indicated using asterisks. Error bars denote SD (^*^p < 0.05, ^**^p < 0.01, ^***^p < 0.001). **(B)** RRI changes are correlated with the survival times of both the heart **(a)** and the brain **(b)**. Each circle represents data from one rat. Linear regression lines were plotted to show the correlation between RRI change and ECG duration **(a)**, and between RRI change and CCoh duration **(b)** for all four groups of rats. Significant correlations between RRI change and ECG duration, or between RRI change and CCoh duration are indicated using asterisks (^*^p < 0.05, ^**^p < 0.01, ^***^p < 0.001).

**Figure 6:**
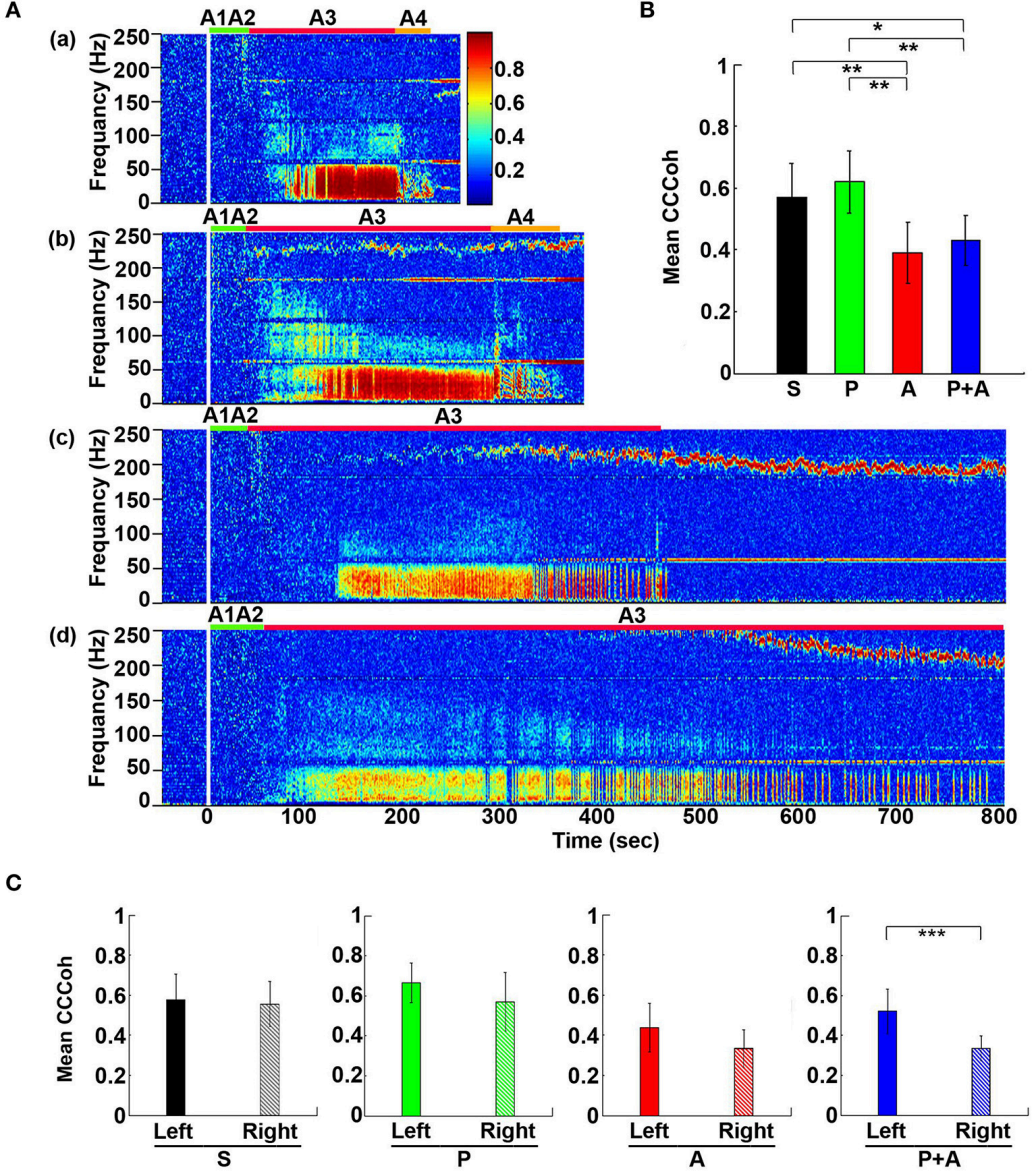
Adrenergic blockade decreases corticocardiac coherence (CCCoh). **(A)** CCCoh (averaged over all channel pairs) before (50 s) and after asphyxia in four groups of rats: **(a)** saline, **(b)** phentolamine, **(c)** atenolol, and **(d)** phentolamine and atenolol. **(B)** The mean and SD of mean CCCoh (5–55 Hz) after asphyxia in four groups of rats. **(C)** Impact of drugs on hemispheric asymmetry of CCCoh in four groups of rats. Significant difference of mean CCCoh among 4 groups of rats **(B)** or between the left and right side of the brain **(C)** are indicated using asterisks. Error bars denote SD (^*^p < 0.05, ^**^p < 0.01, ^***^p < 0.001).

For all of the statistical analyses, Shapiro-Wilk normality test was first implemented to determine if the data was normally distributed. To test the differences of ECG duration (defined as the duration from the asphyxia onset at time 0 until the onset of isoelectric activity; Figure 1B), CCoh duration (Figure 2B), mean CCoh during A3 phase (Figure 3C), RRI change (**Figure 5Ab**), mean CCCoh (**Figure 6B**) and mean CCCon (**Figure 8B**) among 4 groups of rats, as well as the mean CCCoh (**Figure 6B**) and CCCon (**Figure 8B**) among six EEG channels, one way ANOVA with Bonferroni *post hoc* comparisons (for normally distributed data) or Kruskal-Wallis Test with Mann-Whitney *post hoc* comparisons (for non-normally distributed data) were used. To analyze the correlation between ECG duration and CCoh duration (Figure 4), between RRI change and ECG duration (Figure 5Ba), and between RRI change and CCoh duration (Figure 5Bb), Pearson (for normally distributed data) or Spearman (for non-normally distributed data) correlation analysis was performed. For all the comparisons, *p* < 0.05 was considered as statistically significant. Statistical analyses were carried out in consultation with the Center for Statistical Consultation and Research at the University of Michigan. Statistical analyses were performed using the software SPSS (version 19.0; IBM SPSS Statistics).

## Results

### Adrenergic blockade prolongs cardiac electrical activity during asphyxia

Cardiac electrical activity was investigated using ECM to facilitate the visualization of dynamic temporal changes of RRI and cardiac arrhythmias before and after asphyxia (Figure 1A). Raw ECG traces of representative rats were also included for traditional viewing (Figure S1). Before asphyxia, all 4 groups of rats had relatively stable RRI of about 0.2 s with no cardiac arrhythmias. Following the onset of asphyxia, the RRI exhibited dramatic and distinct changes in the four groups of rats. We divided the changes of RRI and cardiac arrhythmias into 5 stages (A1 to A5) according to their cardiac features. Immediately following the asphyxia onset in saline treated rats (Figure 1Aa), RRI began to expand rapidly, from 0.2 s at baseline to 0.6 s at the peak within 10 s (A1 stage), indicating a rapid onset of bradycardia. PR interval lengthened, suggestive of first-degree heartblock. Furthermore, T-waves increased in peak height, and remained high for the entire bradycardia period in all rats. The A1 stage was followed by a transient recovery of RRI that lasted for about 20 s (A2 stage). During this period, two cardiac features are worth noting: elevated T-wave peak height and peak duration and gradually lengthened PR interval. Stages A1 and A2 show similar durations among four groups of rats. The rapid increase of RRI at A1 was reproducibly observed in rats injected with saline (Figure 1Aa) and phentolamine (Figure 1Ab), but was less prominent in rats received atenolol (Figure 1Ac) and phentolamine plus atenolol (Figure 1Ad). T wave elevation was also noted in all 4 groups of rats during the A2 period, but no statistical significance was found between the groups. During this early phase of asphyxia, a variety of cardiac arrhythmias was identified in all rats, which includes premature atrial contraction, premature junctional contraction, premature ventricular contraction, junctional rhythm, and sinus arrhythmias.

In contrast to the A1A2 period, the duration and cardiac arrhythmias in stages A3 and A4 varied significantly. During A3, all rats suffered bradycardia due to 2rd or 3rd degree atrioventricular blocks with sequential junctional and ventricular escape rhythms. RRI was expanded to as long as 1.4 s (Figure 1Ab, stage A3), a 7-fold increase from the baseline values of 0.2 s. This bradycardia period ended in sustained ventricular tachycardia (VT) during A4 and VF during A5 period (not shown in ECM) in rats injected with saline and phentolamine. Interestingly, VT and VF were identified in only 1 of the 8 rats (12.5%) injected with atenolol and 2 of the 11 rats (18.2%) received phentolamine plus atenolol (Table 1). When VT and VF were blocked by these drugs, rats entered an isoelectric stage directly from the A3 stage, exhibiting 3rd degree heart block with ventricular escape beats. This result is consistent with the reported ability of beta blocker to reduce the incidence of ventricular arrhythmias in human patients (Patterson and Lucchesi, 1984; Bourque et al., 2007).

**Table 1:** Summary of ventricular tachycardia and ventricular fibrillation in four groups of asphyxic rats.

	**Saline (*n* = 10)**	**Phentolamine (*n* = 7)**	**Atenolol (*n* = 8)**	**Phentolamine + Atenolol (*n* = 11)**
V. Tachycardia	10/10 (100%)	7/7 (100%)	1/8 (12.5%)	2/11 (18.2%)
V. Fibrillation	10/10 (100%)	7/7 (100%)	1/8 (12.5%)	2/11 (18.2%)

To investigate the effects of adrenergic blockade on cardiac electrical activity, we compared the duration of ECG signals (that begins from the asphyxia onset to the beginning of the isoelectric state) in four groups of rats: saline (305 ± 45 s; mean ± SD), phentolamine (389 ± 72 s), atenolol (532 ± 145 s), and phentolamine plus atenolol (723 ± 304 s). We found a significant increase in the duration of ECG signals in rats received drugs (Figure 1B). Remarkably, rats received phentolamine plus atenolol had the most significant increase in the duration of ECG signals (*p* < 0.001).

### Adrenergic blockade prolongs cortical functional connectivity

Cortical coherence (CCoh) was calculated before and after asphyxia (Figure 2). In all rats, EEG signals displayed intense coherence immediately after asphyxia for high gamma waves (>150 Hz) and for theta waves. In addition, three distinct coherence clusters were noted in all rats: gamma 3 (125–175 Hz), gamma 2 (65–115 Hz), and gamma 1 (25–55 Hz). Of these clusters, gamma 2 and 3 clusters occurred at the transitions between A1 and A2 stages, whereas the gamma 1 cluster occurred at the junction between the A2 and A3 states. The intensity and duration of these coherence clusters did not show significant differences among 4 groups of rats. During the bradycardia period (A3), EEG signals showed intense coherent activity at lower frequency ranges (5–55 Hz) with varying duration and intensity. In rats injected with saline and phentolamine, CCoh was intense and continuous. However, in rats injected with atenolol and phentolamine plus atenolol, CCoh was weaker and intermittent.

To investigate the effects of adrenergic blockade on the length of functional activities of the brain, we compared the duration of CCoh in four groups of rats (Figure 2B): saline (314 ± 45 s), phentolamine (394 ± 61 s), atenolol (492 ± 86 s), and phentolamine plus atenolol (608 ± 127 s). We found significant increase in the duration of CCoh in all three drug groups from the saline treatment. Remarkably, rats received phentolamine plus atenolol had the most significant increase in the duration of CCoh (*p* < 0.001).

### Adrenergic blockade abolishes cardiac event related potentials and cortical coherence during A3 specifically in the right hemisphere

To define the impact of the adrenergic drugs on local neuronal synchrony within the cortex, we analyzed cardiac event related potentials (CERP) (Li et al., 2015a) (Figure 3). EEG potentials (CERP) associated with ECG signals were seen in all rats during A3 phase of asphyxia, although they were undetectable in baseline or at early phase of asphyxia. CERP appeared evenly distributed among the 6 EEG electrodes in the saline treated rats, with slightly higher potential in the occipital lobes compared to the frontal lobes (Figure 3Aa). In the rat treated with both phentolamine and atenolol (Figure 3Ba), however, CERP was severely suppressed in all three electrodes specifically in the right hemisphere. This data demonstrates that adrenergic signaling underlies the CERP and its impact is dominant in the right cerebral hemisphere.

We also separated the coherence pairs formed between electrodes on the left hemisphere from those on the right hemisphere for both control and drug injected rats. Cortical coherence (CCoh) on the left cortex and the right cortex showed similar levels during A3 period (the white dashed boxes in Figure 3Ab) in a control rat. In a marked contrast, the CCoh on the right hemisphere was nearly abolished by the injected adrenergic blockers (Figure 3Bb). The drug-induced hemispheric asymmetry of CCoh during A3 was significant for rats when either atenolol or atenolol and phentolamine was used (Figure 3C). Importantly, when both phentolamine and atenolol were used together, the left and right asymmetry was more significant, compared to when either drug was used alone (Figure 3C). This data indicates that the drugs that do not penetrate the blood-brain-barrier can exert a major impact on the functional connectivity within the cerebral cortex during asphyxic cardiac arrest.

### Brain functional connectivity parallels with cardiac electrical activity

Adrenergic blockade significantly prolonged both cardiac activity (Figure 1) and functional cortical connectivity (Figure 2) and longer survival was observed when both beta1- and alpha-adrenergic receptors were simultaneously inhibited. When ECG duration was compared with CCoh duration (Figure 4), a significance correlation between CCoh duration and ECG duration was found for each group of rats (saline: *r*^2^ = 0.974, *p* < 0.001; phentolalamine: *r*^2^ = 0.969, *p* < 0.001; atenolol: *r*^2^ = 0.731, *p* < 0.05; phentolamine plus atenolol: *r*^2^ = 0.878, *p* < 0.01). A significant correlation was found between CCoh duration and ECG duration (*r*^2^ = 0.983, *p* < 0.001). Thus, the longer the brain was functioning, the longer the heart was signaling, despite a continued lack of oxygen supplies.

### Adrenergic blockade suppresses the initial heart-rate reduction induced by asphyxia

In Figure 1A, we noticed that during A1 and A2, the transient lengthening of RRI (i.e., reduction of heartrate) was less obvious in rats received atenolol and phentolamine plus atenolol than saline and phentolamine. To quantify these changes, we compared the initial rise of RRI in 4 groups of rats (Figure 5). Baseline RRI (RRI^−CO2^) was obtained by averaging the RRI for 100 s-long ECG data before asphyxia induction. RRI after asphyxia (RRI^+CO2^) was defined by the first peak value of RRI after asphyxia. The RRI changes were expressed as percent changes of RRI after asphyxia vs. baseline (RRI^+CO2^*^^100/RRI^−CO2^). We found that the initial increase of RRI was significantly suppressed by each drug (Figure 5Ab), and that rats received both phentolamine and atenolol had the most significant suppression for RRI. When the extent of the RRI changes was compared with drug-dependent changes of ECG duration (Figure 5Ba) and CCoh duration (Figure 5Bb), significant correlations were found between RRI changes and ECG duration (*r*^2^ = −0.689, *p* < 0.01), and between RRI changes and CCoh duration (*r*^2^ = −0.747, *p* < 0.01). These data demonstrate that a larger initial RRI expansion (thus a larger reduction of heart rate) was significantly associated with a shorter ECG duration and a shorter period of brain function.

### Adrenergic blockers suppress brain-heart communication

To explore the effects of adrenergic blockade on functional coupling between the brain and heart, we examined corticocardiac coherence (CCCoh) before and after asphyxia for all four groups of rats (Figure 6), a method developed in our previous study (Li et al., 2015a). CCCoh was not detectable before asphyxia and during stages A1 and A2 in any of the rats. During bradycardia phase (A3), strong CCCoh at lower frequency (5–55 Hz) emerged with a delay, surged to its peak within 2 min of asphyxia, and persisted for as long as the ECG signal was detectable (Figure 6A). Similar to the CCoh examined earlier (Figure 2), CCCoh displayed different durations and distinct patterns among the different drug groups. In rats injected with saline and phentolamine, the duration of CCCoh was short, but the CCCoh was strong and continuous. However, in rats injected with atenolol and phentolamine plus atenolol, the duration of CCCoh was longer, but the CCCoh was weaker and intermittent.

To quantify the effects of adrenergic blockade on the functional coupling between the brain and the heart after asphyxia, we compared the mean intensity of CCCoh in four groups of rats (Figure 6B): saline (0.52 ± 0.068), phentolamine (0.55 ± 0.075), atenolol (0.38 ± 0.095), and phentolamine plus atenolol (0.41 ± 0.063). Since only a few of the rats treated with atenolol or atenolol with phentolamine exhibited ventricular tachycardia/fibrillation at the end of their lives (Table 1), we focused on the A3 period in all subsequent analysis. We found that the mean CCCoh levels was significantly lower in rats received either atenolol or phentolamine plus atenolol than saline or phentolamine alone. This data suggests that beta-adrenergic signaling plays a major role in mediating the brain and heart functional connectivity during asphyxic cardiac arrest. Interestingly, drug-dependent suppression of CCCoh was significantly higher on the right hemisphere, compared to the left when rats received both alpha and beta-blockers (Figure 6C).

### Adrenergic blockade results in marked asymmetry and regional specificity of heart-brain coupling

**Figure 7:**
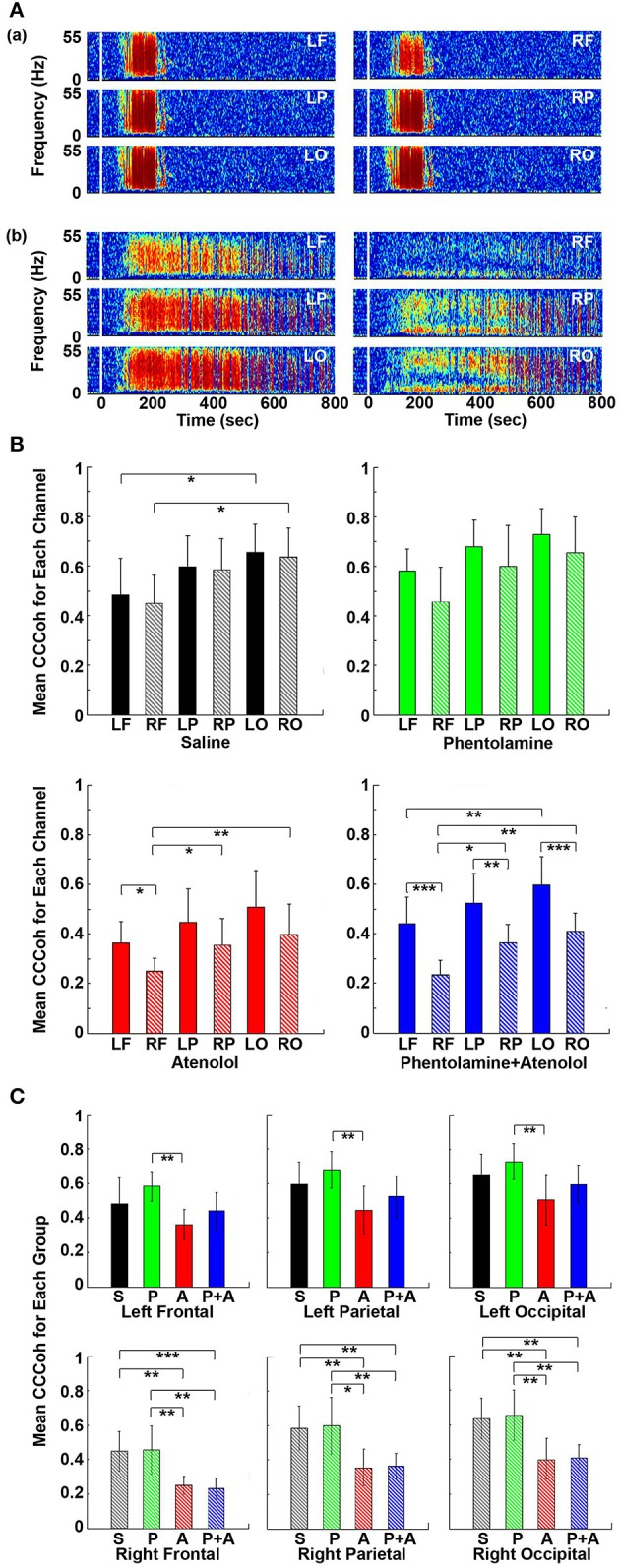
Adrenergic blockade leads to asymmetric corticocardiac coherence (CCCoh). **(A)** CCCoh raw data for each of the six EEG channels rat before (50 s) and after asphyxia is shown for one representative control **(a)** and a rat received phentolamine plus atenolol **(b)**. LF: left frontal; RF: right frontal; LP: left parietal; RP: right parietal; LO: left occipital; RO: right occipital. **(B)** CCCoh displays hemispheric asymmetry in rats treated with drugs. The mean and SD of CCCoh (5–55 Hz) after asphyxia for each of the six EEG channels in four groups of rats. **(C)** Beta blocker inhibits CCCoh specifically on the right hemisphere. Significant differences of mean CCCoh among 6 EEG channels are indicated using asterisks in both **(B,C)**. Error bars denote SD (^*^p < 0.05, ^**^p < 0.01, ^***^p < 0.001).

To further investigate the impact of adrenergic blockers on electrical coupling of the heart with the 6 different regions of the cortex, we examined the coherence between ECG signals and each of the six cortical signals (Figure 7). In rats received saline (Figure 7Aa), CCCoh appeared equally strong at all brain sites and there were no apparent differences between the six cortical regions. In contrast, however, in rats received both phentolamine and atenolol (Figure 7Ab), the right side of the cortex displayed marked reduction of CCCoh compared with the left side. Furthermore, there appeared to be a front-to-back gradient of CCCoh within the cortex, with frontal lobes exhibiting much weaker coupling with the heart than the occipital lobes (Figure 7A).

We quantified the differences in CCCoh among six cortical channels for each of the four groups of rats in A3 phase (Figure 7B). In general, the left side of the cortex appeared to have a stronger coupling with the heart than the right side of the brain, and the frontal lobes displaying lower coupling with the heart than the parietal and occipital lobes. In control rats, the CCCoh in occipital lobes was significantly higher than ipsilateral frontal lobes on both left and right hemispheres. In rats received atenolol, left frontal lobe shows higher CCCoh than the right frontal lobe, which shows lower CCCoh than both the parietal and occipital lobes of the same side. Remarkably, in rats received both phentolamine and atenolol, the CCCoh on the right side of the brain was significantly weaker than the left side of the brain, and the frontal lobe was significantly lower than parietal lobe, which was significantly lower than the occipital lobe. We also compared the CCCoh at each locus between the 4 groups of rats (Figure 7C). Significant differences on the left hemisphere between drug groups were identified only between rats treated with phentolamine and atenolol. This effect appears to result from a slight increase of CCCoh by phentolamine and slight decrease by atenolol, though none of the drug treated groups had significant difference when compared with the control group (upper panels in Figure 7C). On the right hemisphere (lower panels in Figure 7C), however, beta-blockade significantly suppressed CCCoh compared to both control as well as alpha blocker-treated rats. The group treated with both alpha and beta blockers showed identical results with those treated with beta blocker alone, suggesting that alpha blocker had no effect for brain-heart coherence on the right hemisphere. Thus beta-blocker suppresses brain-heart coherence specifically and significantly on the right hemisphere of the brain.

### Beta blocker suppresses bi-directional effective communications between the brain and the heart

**Figure 8:**
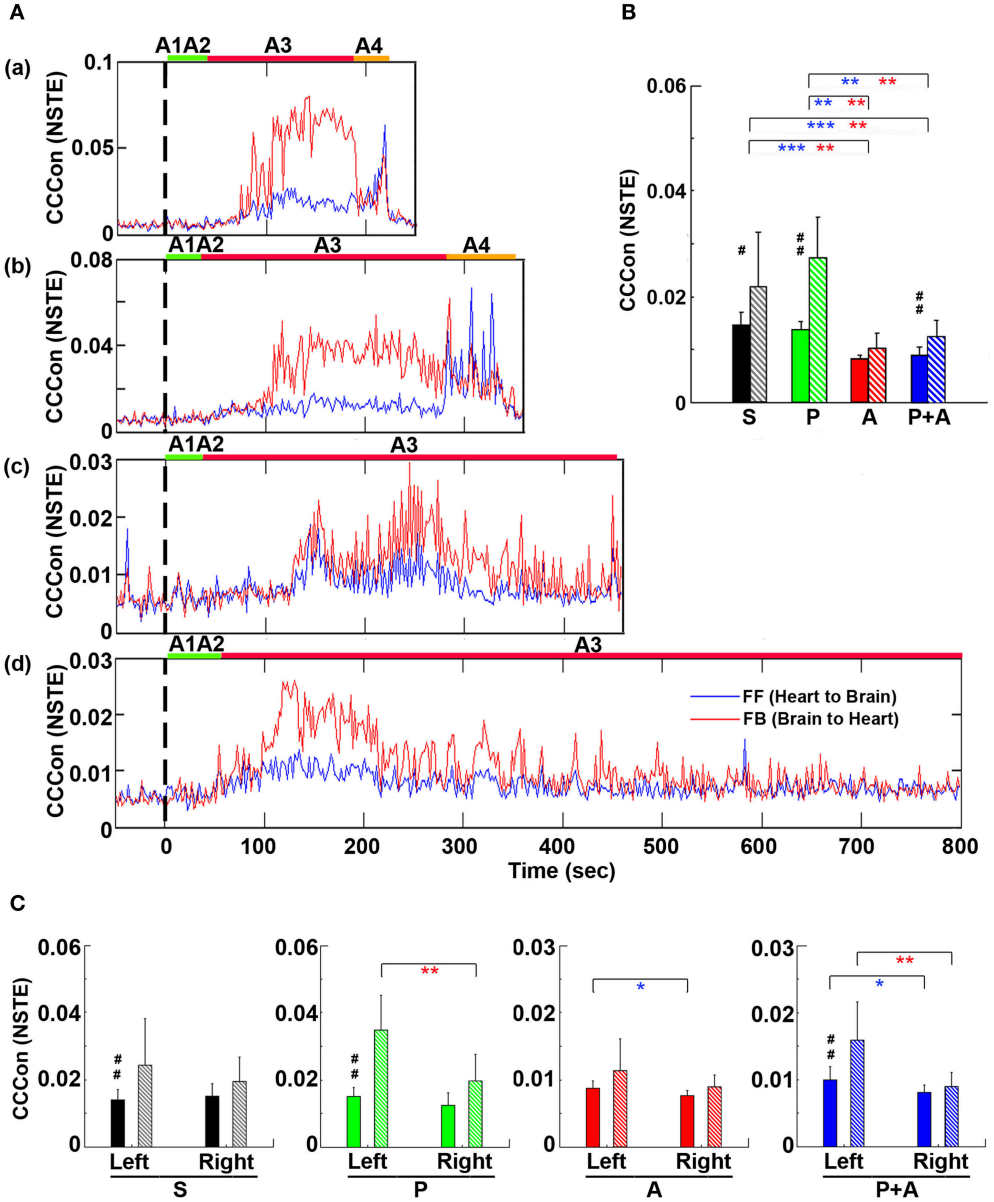
Adrenergic blockade decreases corticocardiac directional connectivity (CCCon). **(A)** CCCon, measured by the Normalized Symbolic Transfer Entropy (NSTE), shows high levels of feedback (from the brain to the heart or efferent) connectivity directed from the brain to the heart in 4 representative rats. CCCon (averaged over six EEG channels) before (50 s) and after asphyxia in four representative rats: **(a)** saline, **(b)** phentolamine, **(c)** atenolol, and **(d)** phentolamine and atenolol. Red trace shows feedforward (from the brain to the heart) CCCon and blue trace shows feedback (from the heart to the brain) CCCon. **(B)** The mean and SD of CCCon (5–55 Hz) during A3 phase (see **A**) in four groups of rats. Solid bars represent afferent CCCon and patterned bars represent efferent CCCon. Significant differences of afferent CCCon among 4 groups of rats are indicated using blue asterisks, and that of efferent CCCon among 4 groups are indicated using red asterisks. **(C)** Left and right asymmetry of afferent and efferent brain-heart connection in different drug groups. Significant differences between afferent and efferent CCCon are marked by pound signs. Error bars denote SD (^*^,#*p* < 0.05, ^**^,##*p* < 0.01, ^***^p < 0.001).

To explore the impact of adrenergic blockade on bi-directional brain-heart information transfer, we examined effective connectivity between the brain and heart (CCCon) in the four groups of rats (Figure 8). In all rats (Figures 8Aa–d), connectivity remained at baseline levels during stages A1 and A2, the early phase of asphyxia. During bradycardia (A3), there was a delayed surge of connectivity in both afferent (feedforward or FF, from the heart to the brain) and efferent (feedback or FB, from the brain to the heart) directions, with efferent connectivity dominated in all rats (Figure 8A).

To quantify the effects of adrenergic blockade on directional information transfer between the brain and the heart following asphyxia, we examined brain-heart effective connectivity in both directions (Figure 8B). The mean CCCon was calculated in four groups of rats in both directions: saline (FF: 0.0159 ± 0.0020; FB: 0.0215 ± 0.0078), phentolamine (FF: 0.0148 ± 0.0017; FB: 0.0250 ± 0.0057), atenolol (FF: 0.0083 ± 0.0009; FB: 0.0101 ± 0.0029), and phentolamine plus atenolol (FF: 0.0090 ± 0.0012; FB: 0.0121 ± 0.0025). We found that the mean CCCon in both directions was significantly lower in rats received atenolol or both phentolamine plus atenolol than saline or phentolamine alone, indicating that blockade of beta-adrenergic signaling significantly decreased the brain and heart bi-directional information transfer during asphyxic cardiac arrest. Furthermore, phentolamine, when combined with atenolol, increased the efferent connectivity, but had no impact on the afferent connectivity, compared with atenolol alone where there was no feedback dominance of the brain-heart connectivity. Further analysis identified the impact of adrenergic drugs on the hemispheric asymmetry of brain-heart connectivity (Figure 8C): In rats treated with atenolol, afferent connectivity (FF-CCCon) is stronger on the left side than on the right side, while in rats treated with phentolamine and phentolamine plus atenolol, efferent connectivity (FB-CCCon) on the right side of the brain is significantly suppressed than on the left side. Moreover, efferent connectivity was significantly stronger than afferent connectivity for control rats as well as rats injected with phentolamine or phentolamine plus atenolol and this effect is significant only on the left hemisphere. The alpha blocker elevated the efferent connectivity specifically on the left hemisphere when injected together with atenolol.

### Adrenergic blockade leads to marked hemispheric asymmetry and regional specificity of heart-brain effective connectivity

**Figure 9:**
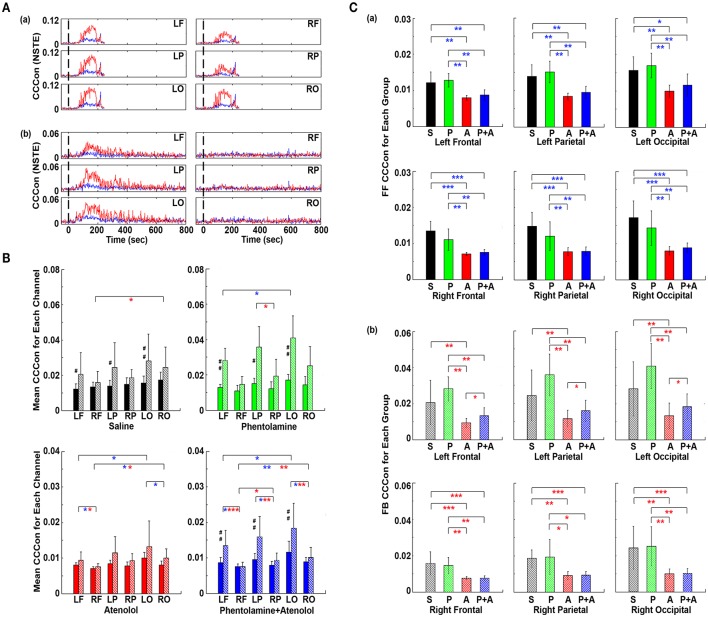
Adrenergic blockade affects corticocardiac connectivity (CCCon) with left and right hemispheric asymmetry, directional asymmetry, and regional specificity. **(A)** CCCon for each of the six EEG channels before (50 s) and after asphyxia in one representative control rat **(a)** and a rat received phentolamine plus atenolol **(b)**. Red trace shows afferent CCCon and blue trace shows efferent CCCon. **(B)** Regional differences on the effect of drugs on CCCon within each drug group. **(C)** Impact of drugs on hemispheric asymmetry, directional asymmetry, and regional specificity of CCCon compared between different drug groups. Solid bars represent afferent CCCon and patterned bars represent efferent CCCon. Significant differences in afferent CCCon among 4 groups of rats are indicated using blue asterisks, while that of efferent CCCon are indicated by the red asterisks. Significant differences between FF and FB CCCon are marked by pound signs. Error bars denote SD (^*^,#*p* < 0.05, ^**^,##*p* < 0.01, ^***^p < 0.001).

To investigate if adrenergic blockers affected brain-heart directional information transfer equally at the left and right, and at the front and back of the brain, we examined the effective connectivity between the heart and each of the six cortical loci (Figure 9). In rats received saline, the connectivity with the heart appeared slightly lower on the right side than the left side of the brain (Figure 9Aa). However, in rats received both phentolamine and atenolol, the connectivity on the right side of the cortex was markedly reduced (Figure 9Ab). Moreover, the connectivity in the frontal lobe appeared lower compared to the parietal and occipital lobes. In both rats, efferent connectivity (red tracings) appeared stronger than afferent connectivity (blue tracings).

Examination of the differences of brain-heart connectivity among six cortical channels for all four groups of rats revealed that the connectivity was affected by the drugs differentially at left and right side, and at the frontal and occipital regions of the brain (Figure 9B). Within each drug group and at each cortical location, connectivity is higher in the efferent direction from the left hemisphere than in the afferent direction, except the atenolol group. There was no directional asymmetry on the right hemisphere at any of the three cortical loci and within any of the 4 groups. Interestingly, no directional asymmetry was found in rats injected with atenolol at any of the 6 cortical regions. These data suggest that efferent signaling to the heart is more robust than afferent communication during asphyxia. Robust and significant efferent dominance over afferent connection was found at left frontal, left parietal, and left occipital lobes in saline, phentolamine, and phentolamine/atenolol treated rats (*p* < 0.05). In addition, all 4 groups of rats exhibited higher levels of afferent connectivity at occipital lobes than at the frontal lobes. In rats treated with both phentolamine and atenolol, both afferent (*p* < 0.05) and efferent (*p* < 0.01) brain-heart connectivity was suppressed on the right side of the brain.

The adrenergic blockers resulted in directional asymmetry (afferent vs. efferent), hemispheric asymmetry (left vs. right), and regional specificity (frontal vs. parietal vs. occipital) of brain-heart connectivity in the dying rats; and the effect was drug-specific. In each of the 6 cortical locations, atenolol significantly reduced the afferent brain-heart connectivity on both left (*p* < 0.01) and right (*p* < 0.001) hemisphere (Figure 9Ca). Phentolamine had no effects on afferent connectivity to any of the 6 cortical loci. In addition, atenolol significantly suppressed efferent brain-heart connectivity in all 6 cortical regions (*p* < 0.01), while phentolamine displayed no significant impact on efferent connectivity on either hemisphere by itself (Figure 9Cb). In rats treated with both atenolol and phentolamine, however, the efferent connectivity was significantly higher in rats treated with both alpha- and beta-blockers than in rats treated with the beta-blocker alone. Importantly this effect of phentolamine on brain-heart connectivity was limited to the efferent direction and exclusive to the left hemisphere (Figure 9Cb).

### Summary of the findings

Our data demonstrate that (1) simultaneous blockade of alpha- and beta-adrenergic signaling extends durations of both cardiac and cortical functional electrical activities and (2) beta-blocker suppresses bi-directional electrical communications between the heart and all regions of the cerebral cortex during asphyxic cardiac arrest. More specifically, beta blocker (1) nearly eliminated the occurrence of ventricular tachycardia and ventricular fibrillation induced by asphyxia, (2) significantly (*p* < 0.01) suppressed the initial and rapid decline of heart rate, (3) reduced the brain-heart coherence, significantly (*p* < 0.01) only on the right hemisphere, and (4) blocked both the efferent/feedback (brain-heart; *p* < 0.01) and afferent/feedforward (heart-brain; *p* < 0.001) signaling. Alpha blocker, on the other hand, (1) reduced the initial decline of heart rate (*p* < 0.01), (2) prolonged the duration of both cardiac (*p* < 0.05) and cortical (*p* < 0.05) functional electrical activities, and (3) when used in combination with beta blocker, reversed beta blocker-mediated significant suppression of efferent/feedback signaling significantly only on the left hemisphere. Importantly, when both alpha- and beta-adrenergic receptors were simultaneously suppressed, ECG duration as well as cortical coherence duration were both lengthened and the combined drug effects were significantly higher than either drug used alone. Furthermore, the combined blockade of alpha and beta-receptors markedly suppressed cortical coherence, specifically on the right hemisphere.

**Figure 10:**
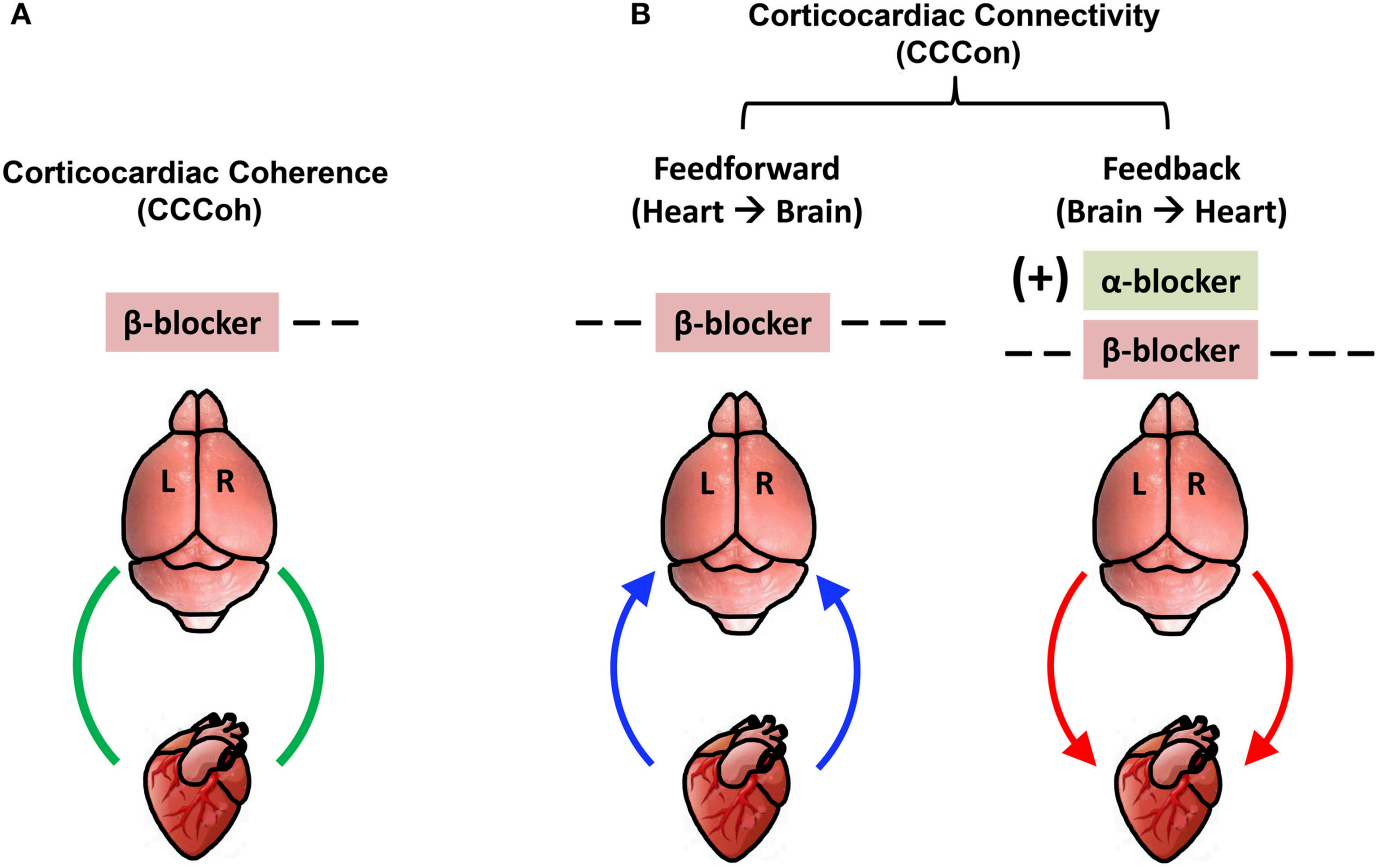
Adrenergic blockers bi-directionally and asymmetrically affect functional brain-heart communication. **(A)** Beta-adrenergic blockade markedly suppresses functional corticocardiac coherence (CCCoh) on the right hemisphere. **(B)** Beta-adrenergic blockade significantly suppresses both the afferent and efferent corticocardiac connectivity (CCCon), especially on the right hemisphere. Alpha-adrenergic blockade increases the efferent CCCon on the left hemisphere in the presence of atenolol. [−−: decrease, *p* < 0.01; −−−: decrease, *p* < 0.001; (+): significant increase only when atenolol was present; L: left; R: right]. The colored curved arrows indicate the direction of brain-heart communication.

The key findings on corticocardiac connectivity are illustrated in Figure 10. The beta blocker atenolol markedly suppressed brain-heart communications in asphyxic rats, by reducing the functional corticocardiac coherence (Figure 10A) and effective corticocardiac connectivity (Figure 10B). It is worth to note that atenolol's inhibitory actions on the brain-heart loop are significantly stronger on the right hemisphere for both coherence (Figure 10A) and connectivity (Figure 10B) measures and that atenolol markedly suppresses both afferent (left panel in B) and efferent (right panel in B) communications between the cortex and the heart. Alpha blocker phentolamine, on the other hand, had no significant effect by itself for brain-heart communication. However when used together with atenolol, phentolamine reversed the suppressive effects of atenolol on brain-heart connectivity. Importantly, this effect was limited to efferent signaling and unique to the left hemisphere (right panel in B). Taken together, these data suggest that beta blocker exerts its beneficial effects by reducing both afferent (feedforward) and efferent (feedback) communications with stronger impact on the right hemisphere.

## Discussion

In this manuscript, we examined the impact of sympathetic blockers on the functional activities of the heart and the brain and functional interaction between the two vital organs in rats during asphyxic cardiac arrest. The results support our hypothesis (Li et al., 2015a) that the rapid cardiac demise by asphyxia stems from the sympathetic insult actively imposed by aroused brain.

### Role of the brain during asphyxic cardiac arrest

Currently, very few studies have focused on the impact of the brain on the heart during cardiac arrest. We have shown that the brain is immediately aroused when rats are exposed to CO_2_ (Li et al., 2015a; this study). The brain activation, reflected by increased cortical coherence at high gamma and theta waves, was associated with an increased RRI (decreased heart rate) and a subsequent brief recovery of RRI within the first minute. Interestingly, there was no detectable electrical communication between the heart and the cortex during this period. A further increase of RRI was followed by a marked surge of brain-heart coherence and connectivity that lasted for the entire duration of ECG signals. These data lead to the following hypotheses: (1) asphyxia-stimulated cortical signaling promotes autonomic activation during early phase of cardiac arrest via a homeostatic survival circuit, and the events in this early phase is likely mediated by subcortical players; (2) when the early rescue efforts fail, the brain mounts an intensive and sustained sympathetic activation, as reflected by the marked surge of corticocardiac coherence and bi-directional corticocardiac connectivity at later phase of cardiac arrest. The fact that adrenergic blockers suppressed the expansion of RRI during the early phase (Figure 5) supports the hypothesis #1 above. The data demonstrating reduced cortex-heart communication by beta-blocker atenolol during the later phase (Figures 7, 9) supports the hypothesis #2. These data suggest that the rapid death by asphyxia is mediated by the overstimulation of the sympathetic system of the brain.

### Animal models for cardiac arrest

Cardiac arrest kills more than 300,000 Americans each year and < 5% of out-of-hospital victims of cardiac arrest survive (Nolan et al., 2012). Initial triggers that lead to cardiac arrest include sudden failures of cardiovascular, neurological, and pulmonary functions, accidents (drowning, choking, automobile accidents, etc.), or drug overdose (Israel, 2014). Most, if not all, of these sudden death occur in the absence of anesthesia. Of the various animal models used for cardiac arrest studies (intra-cardiac injection of toxins, electrical fibrillations, trachea occlusion, and non-oxygen gases induced asphyxiation), however, very few could be performed ethically in the absence of anesthesia. This issue is important, as anesthesia is well known to induce marked changes in autonomic functions (Farber et al., 1995) and can confound the interpretation of studies. Unlike other animal models, CO_2_-mediated asphyxic cardiac arrest can be conducted easily in the absence of anesthesia and has been widely used to euthanize small laboratory rodents. This model has been used successfully to investigate brain-heart interactions during asphyxia (Borovsky et al., 1998; Borjigin et al., 2013; Li et al., 2015a) and was the model of choice for our investigation of brain-heart communications in the present work. In future studies, non-oxygen gases, such as nitrogen (Borovsky et al., 1998), can be tested side-by-side with CO_2_ to examine the impact of hypercapnia on brain-heart coupling. We also plan to monitor additional physiological parameters, such as blood pressure, pulse, and sympathetic nerve activity, in animal models of cardiac arrest.

### Sympathetic toxicity and sudden death

Sudden death is associated with elevated sympathetic activities (Samuels, 2007; Sörös and Hachinski, 2012; Israel, 2014). Elevated efferent sympathetic activity, measured by increased release of plasma norepinephrine, was detected in patients with sleep apnea (Baylor et al., 1995), myocardial damage (Mueller and Ayres, 1980), sustained ventricular arrhythmias (Meredith et al., 1991), and in rats dying from CO_2_ inhalation (Borovsky et al., 1998). Beta blockers reduce incidence of sudden cardiac death, cardiovascular death, and all-cause mortality (Yusuf et al., 1985; Al-Gobari et al., 2013). Atenolol, a blocker of beta-adrenoceptor that does not pass through the blood-brain barrier, is used in human patients to treat a number of conditions including hypertension, angina, acute myocardial infarction, supraventricular tachycardia, and ventricular tachycardia (Patterson and Lucchesi, 1984; Draper et al., 1992). Mechanisms for the beneficial effects of beta-blockers including atenolol, however, remain unclear (Yusuf et al., 1985; Bourque et al., 2007).

CO_2_ results in cardiac arrest in <5 min (Coenen et al., 1995; Li et al., 2015a). When the efferent neuronal signaling was blocked by cord transection, however, the duration of ECG and EEG signals was extended to more than 15 min in asphyxic rats, despite the continued absence of oxygen; and this effect was independent of atropine (Li et al., 2015a). This data suggests that the blockade of sympathetic action may be beneficial for prolonging the survival of both heart and brain in dying individuals, an idea tested in a rat model in the present work. Elevated sympathetic activity was reported in rats dying from CO_2_-mediated asphyxiation (Borovsky et al., 1998). Importantly the effect of CO_2_ on norepinephrine release was shown to result from hypoxic rather than hypercapnic action of the gas and is independent of adrenally released epinephrine (Borovsky et al., 1998). In our studies [Li et al., 2015a; this study], asphyxia induced a delayed surge of corticocardiac coherence and bi-directional connectivity between the brain and the heart. Atenolol, administered prior to asphyxia, significantly suppressed the brain-heart communication (Figures 7–9). In fact, the beta-blocker inhibits both efferent signaling from the brain to the heart and afferent signaling from the heart to the brain and extended the functional activity of both viral organs. In support of our finding, beta-blockers were shown to lower norepinephrine release in human subjects (Mueller and Ayres, 1980; Vincent et al., 1984; Packer, 1998) and rats (Berg, 2014). Thus peripherally acting beta-blocker alters the feedback/efferent effective corticocardiac connectivity by inhibition of norepinephrine release at the presynaptic beta1-adrenoceptors (Berg, 2014). These data indicate that the cerebral cortex is an essential part of a survival feedback loop and support the notion that sympathetic storm stimulated by a sudden drop of cardiovascular functions is the root cause of most, if not all, sudden cardiac arrest cases.

### Cerebral asymmetry in autonomic control of the heart

Brain control of cardiac activity is lateralized, with left hemisphere associated with parasympathetic and right hemisphere with sympathetic functions (Wittling et al., 1998a,b; Critchley et al., 2005; Foster and Harrison, 2006). Consistent with the right hemisphere predominance in sympathetically mediated cardiac control (Wittling et al., 1998b), the beta-blocker specifically and significantly inhibited cortical coherence (Figure 3; in combination with phentolamine) and corticocardiac coherence (Figures 6, 7) only on the right cerebral loci and inhibition of feedforward (afferent) corticocardiac directed connectivity by atenolol was more significant on the right hemisphere (*p* < 0.001) than the left hemisphere (*p* < 0.01) (Figure 9C). Interestingly, atenolol's suppressive effect on brain-heart coupling was not limited to the right hemisphere (Figure 9C); a significant (*p* < 0.01) inhibition of bi-directional effective brain-heart connectivity was detected on the left cortex. This data suggests that blockade of peripheral beta1-adrenergic receptors leads to downregulation of central sympathetic as well as parasympathetic signaling. These data are consistent with known mechanisms of autonomic regulation of the heart (Wehrwein et al., 2016). Furthermore, they support the validity of our new approach for pharmacological dissection of signaling mechanisms within the cardiac survival circuit, to which the cerebral cortex is an essential player.

### Dual alpha and beta blockers for lengthening the functional brain and heart activity

The two branches of the autonomic nervous system regulate cardiac functions through norepinephrine released by the sympathetic nerves and acetylcholine secreted by the vagal nerves. An extensive and reciprocal interaction, reflected by a prejunctional cholinergic modulation of adrenergic (Vanhoutte and Levy, 1980) and prejunctional adrenergic modulation of cholinergic (Akiyama and Yamazaki, 2000) neurotransmissions, exists between the two systems (Vizi, 1974; Vanhoutte and Levy, 1980; Wehrwein et al., 2016). Specifically, norepinephrine, acting on the presynaptic adrenergic receptors inhibits acetylcholine release on postganglionic vagal nerve terminals, and this effect is abolished with phentolamine (Akiyama and Yamazaki, 2000). In the present study, phentolamine, when used together with atenolol, had significantly more beneficial effects in prolonging functional activities of the brain and heart than either drug alone (Figures 1, 2). The dual blockers significantly (*p* < 0.001) increased the duration of functional activities of both the brain and the heart, and suppressed the initial expansion of RRI. Interestingly, the effect of the alpha blocker on brain-heart coupling was limited to the left cerebral hemisphere, in contrast to the right dominance of atenolol's effect. Furthermore, phentolamine's effect was more significant when both drugs were used together (Figures 3B,C, 6C, 7B, 9B), and phentolamine significantly reversed the suppressive effect of atenolol on efferent corticocardiac connectivity, specifically on the left hemisphere (Figure 9Cb). These data suggest that phentolamine acts on the prejunctional alpha adrenoceptors to elevate the release of acetylcholine from cholinergic terminals in the myocardium, when beta1-adrenoceptors are blocked by atenolol.

### Peripheral sympathetic blockade alters cortical and corticocardiac connectivity

Drugs used in this study, atenolol and phentolamine, are well known to affect cardiac functions. Consistent with their roles in the heart, the blockers (1) suppressed occurrence of VT/VF (Table 1 and Figure S1), (2) suppressed the initial drop of heart rate (Figure 5Ab), and (3) extended cardiac survival time (or the ECG duration; Figure 1B). More importantly, although having a minimum blood-brain barrier penetration (Neil-Dwyer et al., 1981; Nordling et al., 1981), they exerted marked impact on the dynamics of cortical functional connectivity (Figure 3) and corticocardiac coupling (Figures 6–9). We believe that this effect is due to the existence of a powerful corticocardiac loop (CCL), an extension of the autonomic nervous system that connects the heart with the cerebral cortex. Changes at either end of the CCL, in the heart or the cortex, can functionally influence the outcome at the other end. Thus, emotional trauma, a largely cerebral event, can precipitate a sudden arrest of the heart (Kassim et al., 2008; Sharkey et al., 2011), possibly via the ***efferent*** branch of this interconnected CCL. Our data in this study provide evidence for the existence of the ***afferent*** branch of the CCL: blocking adrenergic receptors of the heart with drugs that do not penetrate the blood-brain barrier can powerfully alter the functional dynamics of cortical and corticocardiac connectivity. Further investigation of this intriguing loop may help elucidate (1) the role of the brain in cardiac diseases and (2) the impact of cardiac events on brain function. It should be noted that our finding that the functional connectivity within the brain and between the brain and heart is pharmacologically sensitive and hemispherically asymmetric alleviates the concern that ECG signals were artefactually detected in cortical electrodes.

### A new tool for non-invasive investigation of brain-heart communications

Functional communications within the brain between two or more neuronal networks have been studied successfully using coherence (Sakkalis, 2011; Fries, 2015; Harris and Gordon, 2015) and connectivity metrics (Lee et al., 2009, 2015; Li et al., 2015a). Similar methods have been developed for analysis of electrical signals encoding different forms of information. Coherence between EMG and EEG signals, for instance, has been analyzed as a measure for neural control of locomotion (Enders and Nigg, 2016). We have pioneered the use of functional and effective connectivity measures to investigate neural control of cardiovascular functions (Li et al., 2015a). Using this method, we have analyzed coherence and connectivity between heart and brain electrical signals and successfully demonstrated a surprisingly tight electrical communication between the heart and the cortex in dying rats (Li et al., 2015a). While this mode of communication was undetected in healthy rats from the 6 cortical loci, it should be detectable in normal individuals when EEG electrodes are placed directly within brain regions with known involvement in the autonomic control of cardiac functions such as insular cortex, paraventricular nucleus, etc. Our ongoing research in human patients also identified a surge of corticocardiac coherence and connectivity when their cardiac functions failed suddenly (manuscript in preparation). In the present manuscript, we have further expanded the utility of this novel approach to probing the signaling mechanisms of bi-directional brain-heart communication. We plan to apply this approach extensively in future studies to probe the mechanisms of various cardiogenic drugs currently used in clinic.

## Author contributions

JB conceived the project and planned experiments. FT, TL, and GX: Conducted experiments; FT, GX, DL, TG, TS, and AS: Analyzed data; GX: Constructed the graphical user interface for ECM analysis; PF: Assisted with validation of cardiac arrhythmias; FT, MW, and JB: Wrote the paper; All authors approved the final version of the manuscript and agreed to be accountable for all aspects of the work in ensuing that questions related to the accuracy or integrity of any part of the work are appropriately investigated and resolved.

### Conflict of interest statement

The authors declare that the research was conducted in the absence of any commercial or financial relationships that could be construed as a potential conflict of interest.
