# Supplementary material for: Adrenergic Blockade Bi-directionally and Asymmetrically Alters Functional Brain-Heart Communication and Prolongs Electrical Activities of the Brain and Heart during Asphyxic Cardiac Arrest
Source: Front Physiol. 2018 Feb 13;9:99. doi: 10.3389/fphys.2018.00099 (PMC5816970; doi:10.3389/fphys.2018.00099)
Supplement: Supplementary file 1 [file Image1.pdf]

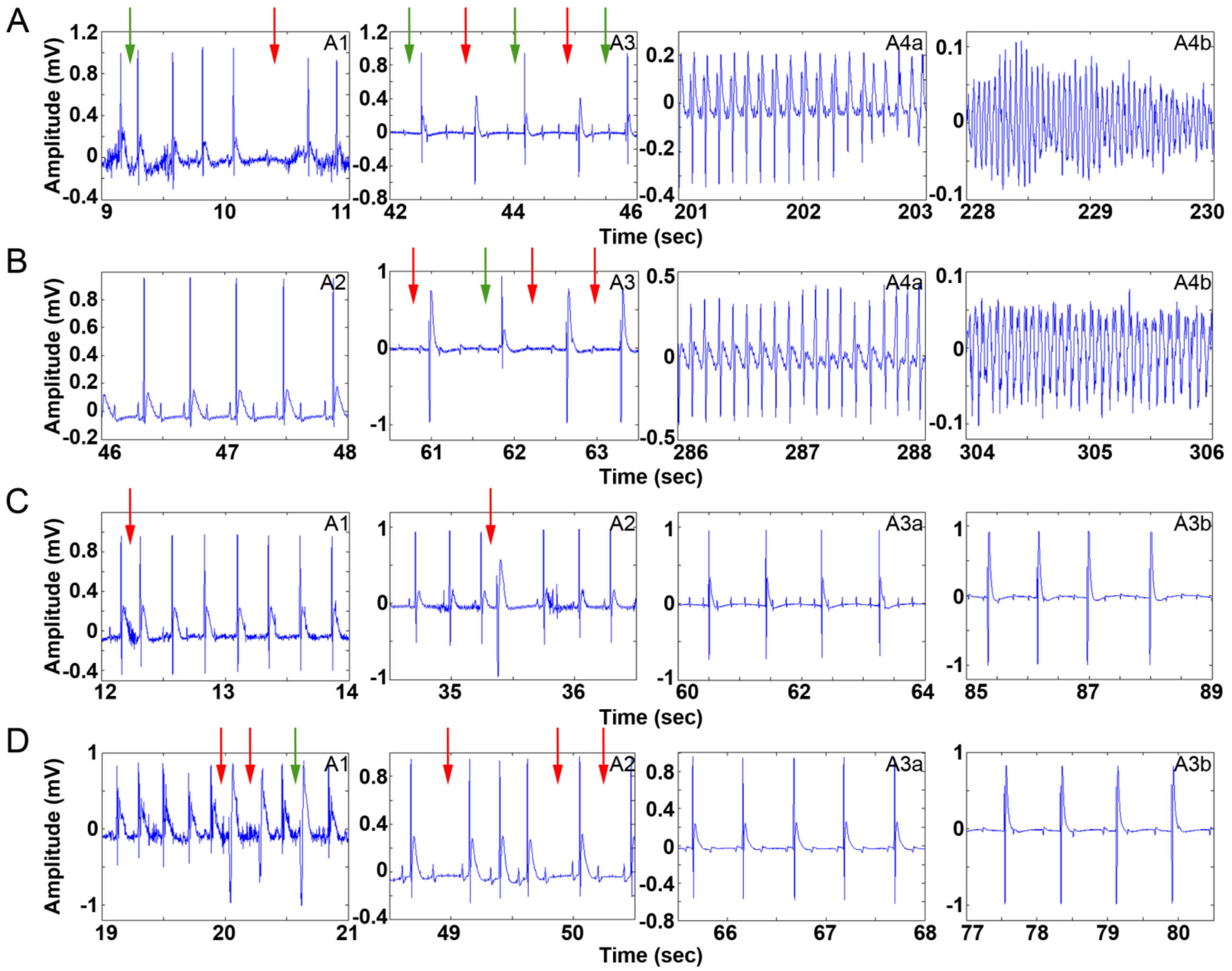

Figure S1. ECG signals with representative cardiac arrhythmias in each stage of asphyxic cardiac arrest for 4 groups of rats. (A) Saline group. A1: 1 premature atria contraction (green arrow) and 1 first-degree heart block (red arrow); A3: 3 junctional escape beat (green arrow) and 2 ventricular escape beat (red arrow) in a background of third-degree heart block; A4a: ventricular tachycardia; A4b: ventricular fibrillation. **(B)** Phentolamine group. A2: second-degree heart block type II; A3: 1 junctional escape beat (green arrow) and 3 ventricular escape beat (red arrow) in a background of third-degree heart block; A4a: ventricular tachycardia; A4b: ventricular fibrillation. **(C)** Atenolol group. A1: 1 premature junctional contraction (red arrow) in a background of junctional rhythm; A2: 1 premature ventricular contraction (red arrow); A3a: junctional escape beat in a background of third-degree heart block; A3b: ventricular escape beat in a background of third-degree heart block. **(D)** Phentolamine plus atenolol group. A1: 1 premature ventricular contraction (green arrow) and 1 ventricular couplet (red arrows) in a background of peaked T waves; A2: 3 second-degree heart block type II (red arrow); A3a: junctional escape beat in a background of third-degree heart block; A3b: ventricular escape beat in a background of third-degree heart block.
